# A systematic review of the applicability of emergency department assessment of chest pain score‐accelerated diagnostic protocol for risk stratification of patients with chest pain

**DOI:** 10.1002/clc.24126

**Published:** 2023-08-18

**Authors:** Minghu Wang, Zhiwei Hu, Lihui Miao, Manman Shi, Qiang Gao

**Affiliations:** ^1^ Emergency Department Beijing Rehabilitation Hospital of Capital Medical University Beijing China; ^2^ School of Acupuncture‐Moxibustion and Tuina Beijing University of Chinese Medicine Beijing China; ^3^ Department of Gastroenterology and Hepatology Beijing Rehabilitation Hospital of Capital Medical University Beijing China

**Keywords:** emergency department (ED), emergency department assessment of chest pain score‐accelerated diagnostic protocol (EDACS‐ADP), major adverse cardiovascular events (MACE), systematic review

## Abstract

The emergency department assessment of chest pain score‐accelerated diagnostic protocol (EDACS‐ADP) are commonly used for risk stratification in undifferentiated patients with acute chest pain. This systematic review aimed to investigate EDACS‐ADP for risk stratification of emergency department (ED) patients with chest pain. The PubMed, Web of Science, Medline, Cochrane Library, China National Knowledge Infrastructure, and Wanfang databases were searched for related studies without restrictions on the publication year. The Quality Assessment of Diagnostic Accuracy Studies 2 tool was used to assess the risk of bias, and Stata 16.0 was used to determine the combined sensitivity, specificity, positive diagnostic likelihood ratio (DLR), and negative DLR. Twelve studies comprising 14 290 patients were identified. Of these, 7537 (52.74%) patients were considered low risk, and 67 (0.89%) had major adverse cardiovascular events (MACE), including myocardial infarction, stroke, and cardiovascular death within 30 days of the patients' ED presentation. EDACS‐ADP showed a combined sensitivity of 0.97 (95% confidence interval [CI]: 0.95−0.99); specificity, 0.58 (0.53−0.63); positive DLR, 2.34 (2.08−2.63); negative DLR, 0.04 (0.02−0.09); diagnostic odds ratio, 53.11 (26.45−106.63); and summary receiver operating characteristic area under the curve, 0.83 (0.79−0.86). Despite the large statistical heterogeneity of the results, EDACS‐ADP identified a considerable number of low‐risk patients for early discharge, with a specificity >50% and an incidence of MACE within 30‐days of patients' ED presentation <1%. Thus, it is a useful tool with a potential for clinical application.

## Graphical abstract

Emergency department assessment of chest pain score‐accelerated diagnostic protocol (EDACS‐ADP) identified a considerable number of low‐risk patients for early discharge, with sensitivity of 0.97, specificity of 0.58, and an incidence of major adverse cardiovascular events within 30‐days of patients' ED presentation 0.89%. It is a useful tool with a potential for clinical application.

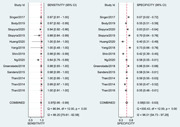

## 1 INTRODUCTION

Chest pain is a common symptom of patients in the emergency department (ED), and acute chest pain accounts for 8%−10% of all ED visits.^1^ Chest pain can be a common symptom of a range of etiologies but is mainly caused by thoracic disorders. Patients with ST‐elevated myocardial infarction can receive a preliminary diagnosis through electrocardiography (ECG); however, other patients with chest pain must undergo further tests and observations for a confirmed diagnosis. Thus, the general recommendation in clinical practice^2^ is to observe the patients for 6−12 hour in cases where high‐risk chest pain cannot be excluded. In this high‐risk group, only 15%−25% of patients are diagnosed with a high‐risk condition, predominantly acute coronary syndrome (ACS). More than 2% of patients with ACS are misdiagnosed in the ED and discharged; their 30‐day mortality risk is nearly twice that of hospitalized patients with ACS.^3^

In recent years, a variety of protocols aimed at rapid risk stratification of patients with acute chest pain have been validated in different studies.^4^ Than et al. replaced thrombolysis in the myocardial infarction score in the 2 hour accelerated diagnostic protocol (ADP) to assess patients with chest pain symptoms using contemporary troponins as the only biomarker (ADAPT‐ADP). This was a part of their newly developed emergency department assessment of chest pain score (EDACS) to develop EDACS‐ADP to rapidly assess patients with acute chest pain and identify high‐risk cases.^5^ Low risk was defined as EDACS <16, 0 and 2 hour troponin levels below the 99th percentile upper reference limit of the assay, and ECG (−), shown as Supporting Information: Table 1. Their findings indicated that 57.6% of the patients with chest pain could be categorized as low‐risk, with a sensitivity of up to 99%^6^; however, the performance of EDACS‐ADP proved unstable in an external cohort. Therefore, we performed a systematic review to analyze the applicability of EDACS‐ADP for risk stratification of patients with chest pain in the ED.

## 2 METHODS

### 2.1 Search strategy

Two researchers searched PubMed, Web of Science, Medline, Cochrane Library, China National Knowledge Infrastructure, and Wanfang databases for randomized controlled trials and cohort studies related to the use of EDACS‐ADP in ED patients with chest pain, without applying filters for the year of publication. Conference abstracts were hand searched and included if they gave sufficient methodological detail to demonstrate they met the inclusion criteria. The following search strategies were implemented:

English databases: #1: chest pains [MeSH Terms]; #2: (Chest Pain [Title/Abstract]) OR (stethalgia [Title/Abstract]) OR (Pectoralgia [Title/Abstract]) OR (Pains, Chest [Title/Abstract]) OR (Thoracalgia [Title/Abstract]) OR (Thoracodynia [Title/Abstract]); #3: (Chest Pain [Text Word]) OR (Stethalgia [Text Word]) OR (Pectoralgia [Text Word]) OR (Pains, Chest [Text Word]) OR (Thoracalgia [Text Word]) OR (Thoracodynia [Text Word]); #4: (EDACS‐ADP [Title/Abstract]) OR (Emergency Department Assessment of Chest Pain Score‐Accelerated Diagnostic Protocol [Title/Abstract]); #5: (EDACS [Title/Abstract]) OR (Emergency Department Assessment of Chest Pain Score [Title/Abstract]); #6: ([Title/Abstract]) OR [Title/Abstract]); #7: #1OR#2OR#3; #8: #5 AND #6; #9: #4 OR #8; #10: #7 AND #9.

Chinese databases: (“EDACS”) OR (“Emergency Department Assessment of Chest Pain Score‐Accelerated Diagnostic Protocol”) OR (“EDACS‐ADP”).

This meta‐analysis was registered in PROSPERO (registration number CRD: 42021269142) and will be reported according to the PRISMA guidelines.^7^

### 2.2 Inclusion and exclusion criteria

The following studies were included: (1) diagnostic studies published from China or internationally on the value of EDACS‐ADP for acute chest pain; (2) studies with 30‐day follow‐up on the outcomes of patients with acute chest pain; (3) major adverse cardiovascular events (MACE) within 30‐days of patients' ED presentation as an outcome event; and (4) studies with complete raw data, such that the true positive (TP), false positive (FP), false negative (FN), and true negative (TN) values of the screening test could be obtained directly or indirectly. The following were the exclusion criteria: (1) no 30‐day follow‐up or outcome results and (2) incomplete data or information.

### 2.3 Data screening and extraction

Two researchers performed literature screening and data extraction independently, and their results were cross‐checked with all discrepancies resolved by a third party. Literature screening first involved removing duplicate studies, followed by reading the article title and abstract to remove clearly unrelated studies. Finally, the researchers read the full article to decide whether to include the study. The data extracted included the study characteristics (title, first author, publication year, country, and gold standard), patient characteristics (age and sex), study setting, study methods, sample size, study quality, outcome measures, and results.

### 2.4 Quality assessment

Using the RevMan 5.3 software (The Cochrane Collaboration), two researchers independently assessed the risk of bias and quality of the studies based on the Quality Assessment of Diagnostic Accuracy Studies 2 (QUADAS‐2) tool.

### 2.5 Data analysis

Stata 16.0 (StatCorp LLC) was used to collate the TP, FP, TN, and FN values for each study and obtain the corresponding combined sensitivity, specificity, positive diagnostic likelihood ratio (DLR), and negative DLR. The heterogeneity among study results was determined using the *χ* ^2^ and *I* ^2^ tests. If considerable heterogeneity was observed, a regression analysis was performed to determine the cause. The summary receiver operating characteristic (SROC) curve was plotted to obtain the area under the curve, the sensitivity analysis graph was used to evaluate result stability, the Deeks' funnel plot was used to assess publication bias, and the Fagan plot was used to assess the clinical value of EDACS‐ADP.

## 3 RESULTS

### 3.1 Included studies

**Figure 1:**
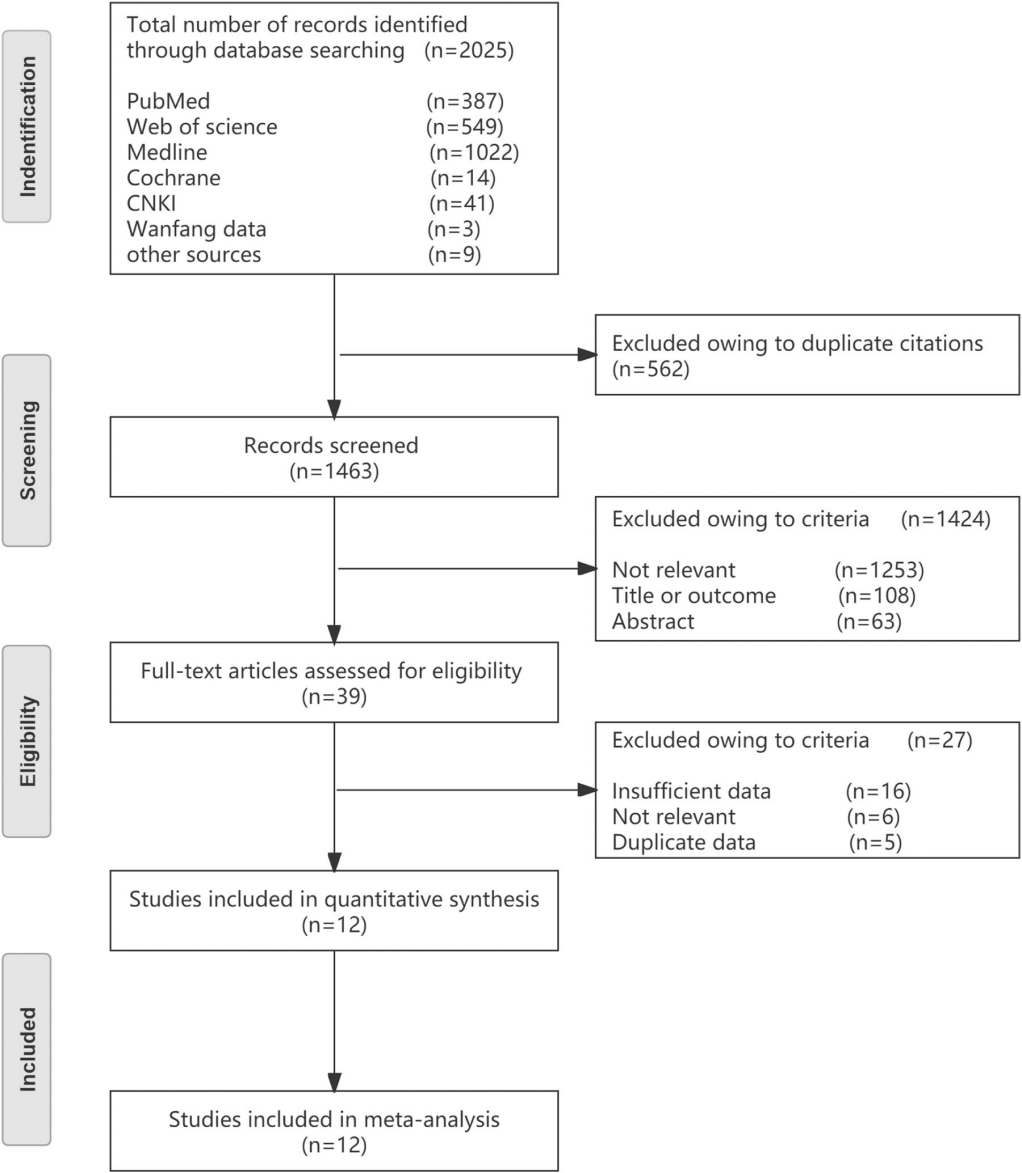
Search strategy for the inclusion of studies in the systematic review and meta‐analysis. CNKI, China National Knowledge Infrastructure.

A total of 2025 studies were identified from the Chinese and English databases, including 44 Chinese studies and 1981 English studies. Among them, 12 studies met the inclusion criteria providing 13 data sets. The literature search process is shown in Figure 1, and details on the studies are shown in Table 1.^5^, ^8^, ^9^, ^10^, ^11^, ^12^, ^13^, ^14^, ^15^, ^16^, ^17^, ^18^

**Table 1:** Basic details of the included studies (12 included studies providing 13 data sets).

Cohort no	First author	Year	Country/region	Sample size (*n*)	Average age (years)	Sex ratio (M/F)	Study design	Outcome measure
1	Stopyra^8^	2015	United States	282	53.3	120/162	Prospective	MACE
2	Than^9^	2016	New Zealand	279	59.6	166/113	Prospective	MACE
3	Ng^10^	2020	Singapore	1195	55.9	817/378	Prospective	MACE
4	Shin^11^	2019	South Korea	1273	62.0	787/486	Retrospective	MACE
5	Than^5^	2014	Australia and New Zealand	1974	60.5	1184/790	Prospective	MACE
6	Than^5^	2014	Australia and New Zealand	608	60.1	359/249	Prospective	MACE
7	Stopyra^12^	2020	United States	4339	54.0	1965/2374	Prospective	MACE
8	Yang^13^	2018	Hong Kong	231	57.6	114/117	Retrospective	MACE
9	Sanders^14^	2015	Australia and New Zealand	909	60.1	538/371	Prospective	MACE
10	Body^15^	2019	Britain	844	Not reported	Not reported	Prospective	MACE
11	Singer^16^	2017	United States	411	Not reported	Not reported	Prospective	AMI
12	Huang^17^	2020	China	134	52.3	82/52	Prospective	AMI
13	Greenslade^18^	2018	Australia	1811	52.9	1086/725	Prospective	AMI

Abbreviations: AMI, acute myocardial infraction; F, female; M, male; MACE, major adverse cardiovascular events.

### 3.2 Quality assessment

The results of the quality assessment based on the QUADAS‐2 criteria (Supporting Information: Figure 1) show that the quality of included studies was high, and the inclusion criteria were satisfied.

### 3.3 Analysis of diagnostic performance

**Figure 2:**
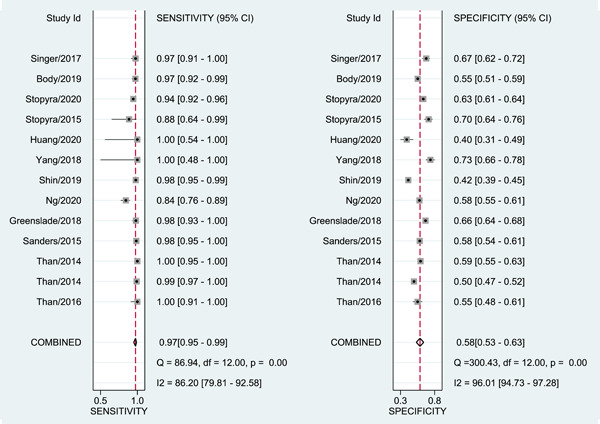
Forest plots for the combined sensitivity and specificity. CI, confidence interval; df, degree of freedom; Q, Cochran's heterogeneity statistic.

**Figure 3:**
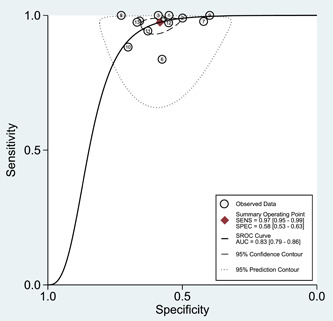
SROC curve for emergency department assessment of chest pain score‐accelerated diagnostic protocol chest pain risk stratification. AUC, area under the curve; SENS, sensitivity; SPEC, specificity; SROC, summary receiver operating characteristic.

Of the 12 studies included in the systematic review, 1671 of 14,290 patients (12%) experienced MACE within 30 days of patients' ED presentation. Based on EDACS‐ADP, 7537 (52.74%) of the pooled patient group were classified as low risk, while 67 (0.89%) patients in the low‐risk group suffered from MACE within 30‐days of patients' ED presentation. The diagnostic accuracy of EDACS‐ADP was 63.5%. Figure 2 shows that the pooled sensitivity of MACE for the 30‐day prediction among low‐risk EDACS‐ADP was 0.97 (95% confidence interval [CI]: 0.95−0.99); the specificity was 0.58 (95% CI: 0.53−0.63). Supporting Information: Figures 2‐3 shows the combined positive DLR, 2.34 (95% CI: 2.08−2.63); negative DLR, 0.04 (95% CI: 0.02−0.09); and diagnostic odds ratio, 53.11 (95% CI: 26.45−106.63). Figure 3 shows the SROC curve for the combined data, the area under the SROC curve was 0.78 (95% CI: 0.74−0.81).

**Figure 4:**
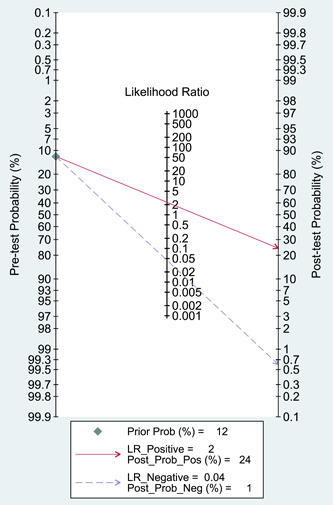
Fagan plot of emergency department assessment of chest pain score‐accelerated diagnostic protocol chest pain risk stratification. LR, likelihood ratio; Neg, negative; Pos, positive; Post, posttest; Prob, probability.

Figure 4 shows the Fagan plot depicting the relationship among the pretest probability, likelihood ratio, and posttest probability. The pretest probability was 12%, and the connected positive DLR was 2, suggesting that after risk stratification with EDACS‐ADP, the posttest probability reached 24%. In addition, the Deeks' funnel plot indicated no publication bias (*p* = .80). Supporting Information: Figure 4 shows the Deeks' funnel plot for EDACS‐ADP chest pain risk stratification.

The *Q* statistics of sensitivity, specificity, positive predictive value, negative predictive value, positive DLR, and negative DLR all reached statistical significance (*p* < .001), with all *I* ^2^ > 75%. These findings indicate a high level of heterogeneity among the studies. A regression analysis was thus conducted, which showed that the source of heterogeneity could have been the publication year, sex, and sample size. Subgroup analyses were performed based on the publication year, study type, country/region, sample size, age, sex, and outcomes. Details of subgroup analyses are shown in Supporting Information: Figure 5. In the subgroup analysis for the year of study publication, studies published before 2015 show a higher sensitivity of 0.99 (95% CI: 0.98−1.00) than studies published after 2015, which had a sensitivity of 0.96 (95% CI: 0.94−0.99). In the subgroup analysis for the study sample sizes, studies that included fewer than 1000 cases showed a higher sensitivity of 0.98 (95% CI: 0.97−1.00). In the subgroup analysis for sex, studies with a male/female ratio of subjects included less than 1 showed a higher specificity of 0.68 (95% CI: 0.61−0.76).

## 4 DISCUSSION

Risk stratification tools for acute chest pain can reduce the rate of misdiagnosis and wrong treatment among high‐risk patients with chest pain in the ED, while enabling the early discharge of low‐risk patients with chest pain. Thus, their prospects for future applications are promising. EDACS‐ADP is a simple and flexible decision aid, which allows clinicians to perform risk stratification of patients with chest pain by combining the patient's troponin results at 0 and 2 hour to determine whether the patient should be admitted or discharged. However, the unstable performance of EDACS‐ADP in external cohorts has consistently been a key factor preventing its clinical application. To the best of our knowledge, Roche et al.^19^ conducted the first meta‐analysis on the diagnostic accuracy of the EDACS‐ADP. In this meta‐analysis, data from 11 studies published between 2011 and 2015 showed that EDACS‐ADP had one of the lowest rates of MACE in those discharged (3/1148, 0.3%) and discharged one of the highest percentages of patients (44.5%) compared to other risk stratification tools such as Goldman, HEART, GRACE, and ADAPT‐ADP. On this basis, we include the most recent update studies for further study. We showed that the sensitivity and the proportion of emergency patients with chest pain classified as low risk in our analysis were lower than those of the original EDACS‐ADP study, whereas our specificity was significantly higher.

As shown in Supporting Information: Table 2, a comparison of other ADPs, including the Asia‐Pacific evaluation of chest pain trial accelerated diagnostic protocol (ASPECT‐ADP)^20^; triage rule‐out using high‐sensitivity troponins accelerated diagnostic protocol (TRUST‐ADP)^21^; the history, ECG, age, risk factors, troponin (HEART) pathway^22^; Vancouver chest pain rule^23^; ADAPT‐ADP^24^; and 2 hour ADP to assess patients with chest pain symptoms using high‐sensitivity troponins as the only biomarker (mADAPT‐ADP)^25^ with EDACS‐ADP revealed that EDACS‐ADP could identify low‐risk patients with a specificity lower than that of the HEART pathway but significantly higher than that of the other ADPs. Essentially, EDACS‐ADP can enable clinicians to get more low‐risk patients discharged earlier. Although it showed the lowest sensitivity, the 30‐day incidence of MACE within 30‐days of patients' ED presentation in low‐risk patients was not significantly higher than that for the other ADPs.

Owing to the enormous variations in the etiologies and disease severity of acute chest pain, as well as the potentially catastrophic consequences of high‐risk chest pain, risk stratification tools for chest pain emphasize sensitivity to avoid a missed diagnosis for patients with high‐risk chest pain to the greatest extent possible, which is a shared consensus among clinicians. A study has shown that a MACE incidence >1% among low‐risk patients was unacceptable to nearly half of the physicians, whereas an incidence >2% was unacceptable to most physicians.^26^ Therefore, when implementing the EDACS‐ADP for the risk stratification of patients with chest pain, clinicians should note that it can identify more low‐risk patients for early discharge, and the risk of missed diagnosis among high‐risk patients is acceptable.

This study had some limitations. First, only the studies published in Chinese and English languages were included; thus, those in other languages were not included, which may have affected our search results. Second, the gold standards used in the included studies were not completely consistent, which may have affected the strength of our results. Third, the statistical analysis revealed significant heterogeneity among the included studies; hence, more clinical studies are required to verify the reliability of our results. Fourth, the external cohort results for EDACS‐ADP were unstable, and the regression analysis identified the study type and country/region as possible sources of heterogeneity. This suggests that heterogeneity is related to the basic information of the enrolled patients. Thus, it is necessary to design larger‐scale prospective studies and reinforce the control of relevant factors. Further investigations should be conducted on the applicative value of EDACS‐ADP in the risk stratification of ED patients with chest pain to obtain more robust data that will enable the wider adoption of this tool among clinicians.

In conclusion, despite the significant heterogeneity detected in the included studies, EDACS‐ADP was able to identify a considerable number of low‐risk patients for early discharge, which can help alleviate ED crowding and reduce the waste of medical resources. Moreover, MACE incidence within 30‐days of patients' ED presentation among low‐risk patients was 0.89%, which was not significantly higher than that for other ADPs. Thus, EDACS‐ADP is efficient for the risk stratification of patients presenting to EDs with chest pain and has an acceptable safety profile.

## AUTHOR CONTRIBUTIONS

Minghu Wang and Zhiwei Hu contributed to the study conception and design, searched the articles, independently reviewed all identified articles for eligibility, performed the data analyses, and wrote the original draft. Lihui Miao and Manman Shi assisted in data acquisition and review literature. Critically revised the work were performed by Qiang Gao. All authors read and approved the final manuscript.

## CONFLICT OF INTEREST STATEMENT

The authors declare no conflict of interest.

## Supporting information


**Supplementary Fig. 1** Assessment of methodological quality of each study using the Quality Assessment of Diagnostic Accuracy Studies 2.

**Supplementary Fig. 2** Forest plots for the combined positive and negative diagnostic likelihood ratios. CI, confidence interval; df, degree of freedom; DLR, diagnostic likelihood ratio; Q, Cochran's heterogeneity statistic.

**Supplementary Fig. 3** Forest plots for the combined diagnostic odds ratio and diagnostic score. CI, confidence interval; df, degree of freedom; Q, Cochran's heterogeneity statistic.

**Supplementary Fig. 4** Deeks’ funnel plot of Emergency Department Assessment of Chest Pain Score‐Accelerated Diagnostic Protocol chest pain risk stratification. ESS, effective sample size.

**Supplementary Fig. 5** Forest plots for univariate meta‐regression and subgroup analyses. CI, confidence interval; I2, I^2^ tests; I2lo, I^2^ tests (low values); I2hi, I^2^ tests (high values); LRTChi2, χ^2^ tests.

Supporting information.

Supporting information.

## Data Availability

The authors confirm that the data supporting the findings of this study are available within the article. The other materials for this study are available to contact with correspondence.
