# Supplementary material for: A systematic review of the applicability of emergency department assessment of chest pain score‐accelerated diagnostic protocol for risk stratification of patients with chest pain
Source: Clin Cardiol. 2023 Aug 18;46(11):1303–9. doi: 10.1002/clc.24126 (PMC10642332; doi:10.1002/clc.24126)
Supplement: Supplementary file 1 — Supplementary Fig. 1 Assessment of methodological quality of each study using the Quality Assessment of Diagnostic Accuracy Studies 2. [file CLC-46-1303-s005.pdf]

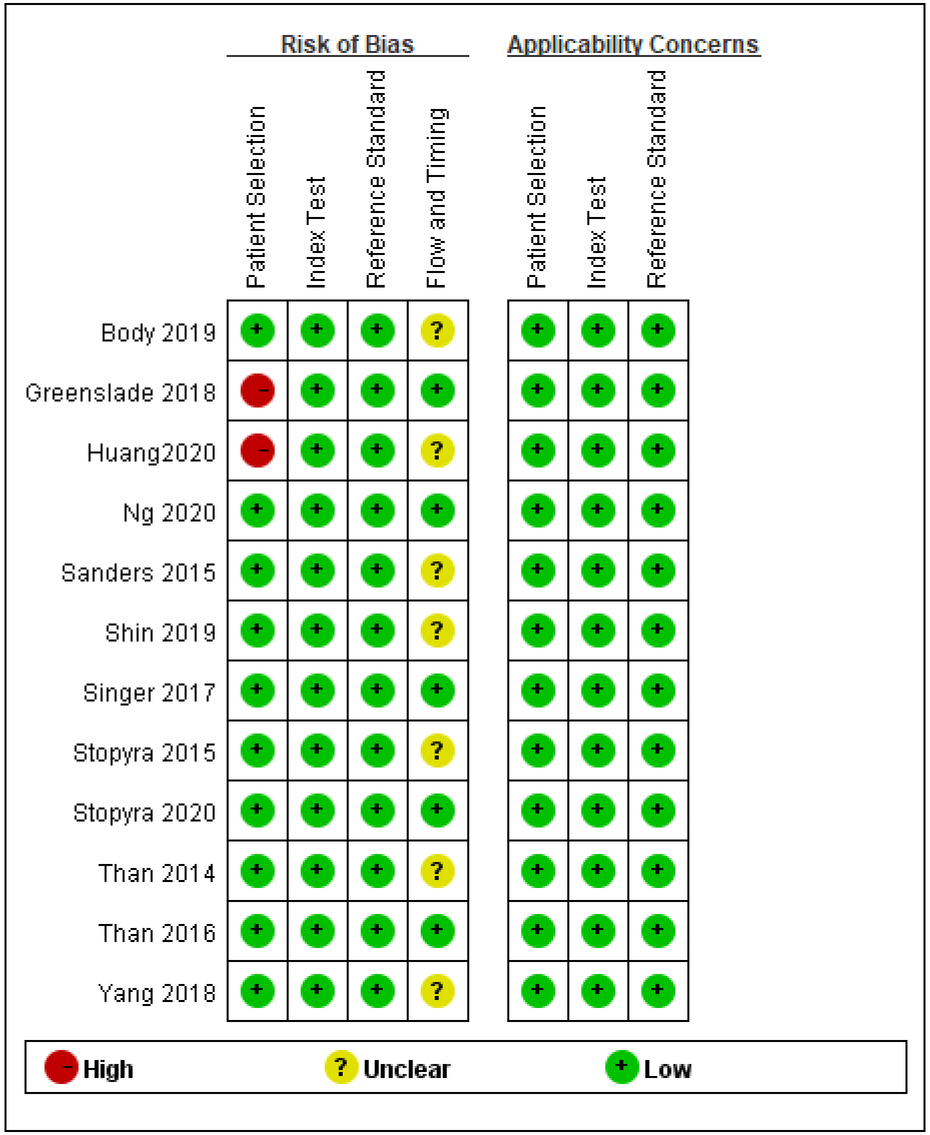

Risk of Bias
Applicability Concerns
Patient Selection
Index Test
Reference Standard
Flow and Timing
Patient Selection
Index Test
Reference Standard
Body 2019
Greenslade 2018
Huang2020
Ng 2020
Sanders 2015
Shin 2019
Singer 2017
Stopyra 2015
Stopyra 2020
Than 2014
Than 2016
Yang 2018
High
Unclear
Low

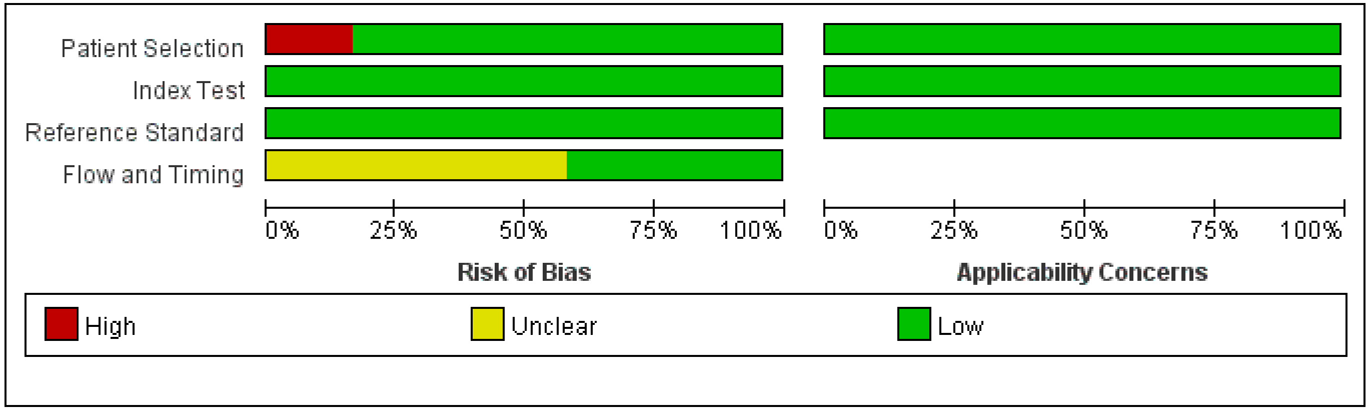
Patient Selection
Index Test
Reference Standard
Flow and Timing
0%
25%
50%
75%
100%
Risk of Bias
Applicability Concerns
High
Unclear
Low
