# Supplementary figures and images for: A systematic review of the applicability of emergency department assessment of chest pain score‐accelerated diagnostic protocol for risk stratification of patients with chest pain
Source: Clin Cardiol. 2023 Aug 18;46(11):1303–9. doi: 10.1002/clc.24126 (PMC10642332; doi:10.1002/clc.24126)

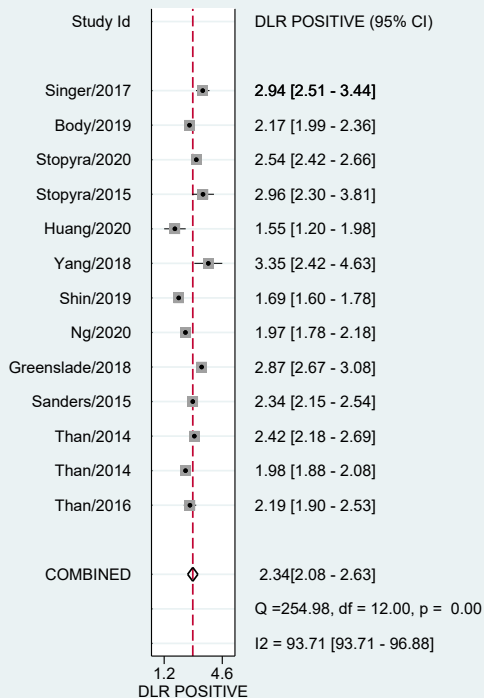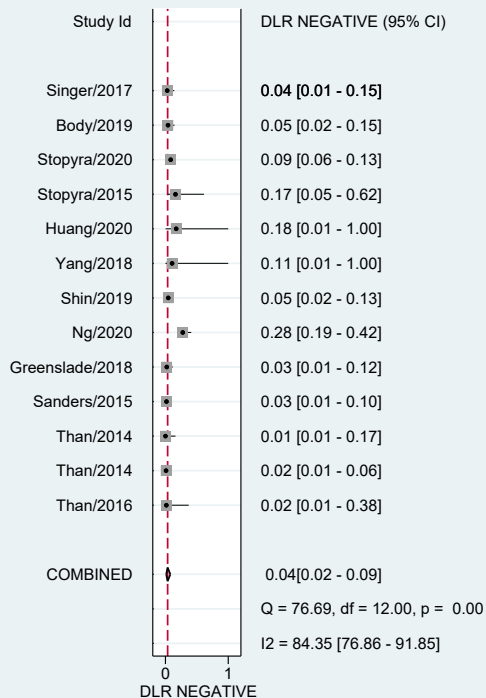

Supplement: Supplementary file 2 — Supplementary Fig. 2 Forest plots for the combined positive and negative diagnostic likelihood ratios. CI, confidence interval; df, degree of freedom; DLR, diagnostic likelihood ratio; Q, Cochran's heterogeneity statistic. [file CLC-46-1303-s001.pdf]

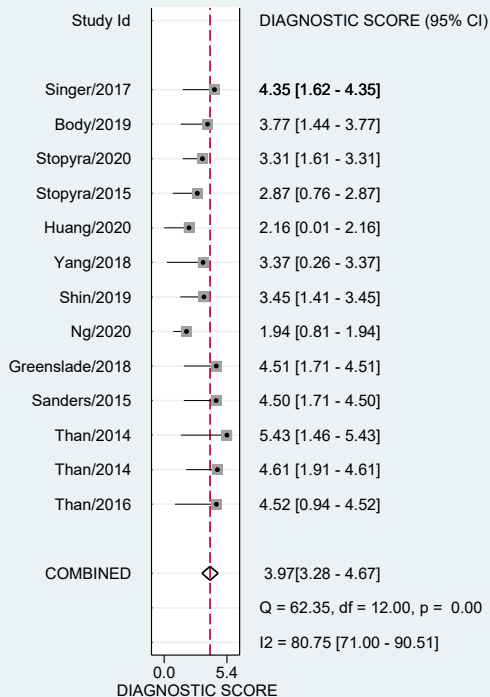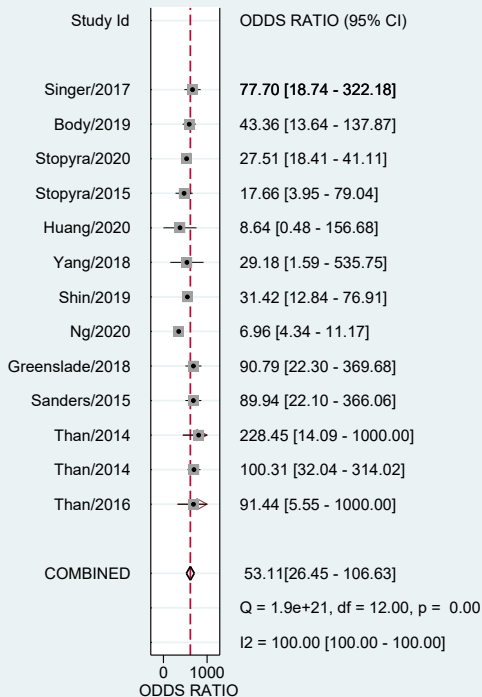

Supplement: Supplementary file 3 — Supplementary Fig. 3 Forest plots for the combined diagnostic odds ratio and diagnostic score. CI, confidence interval; df, degree of freedom; Q, Cochran's heterogeneity statistic. [file CLC-46-1303-s003.pdf]

Deeks' Funnel Plot Asymmetry Test  
pvalue = 0.80

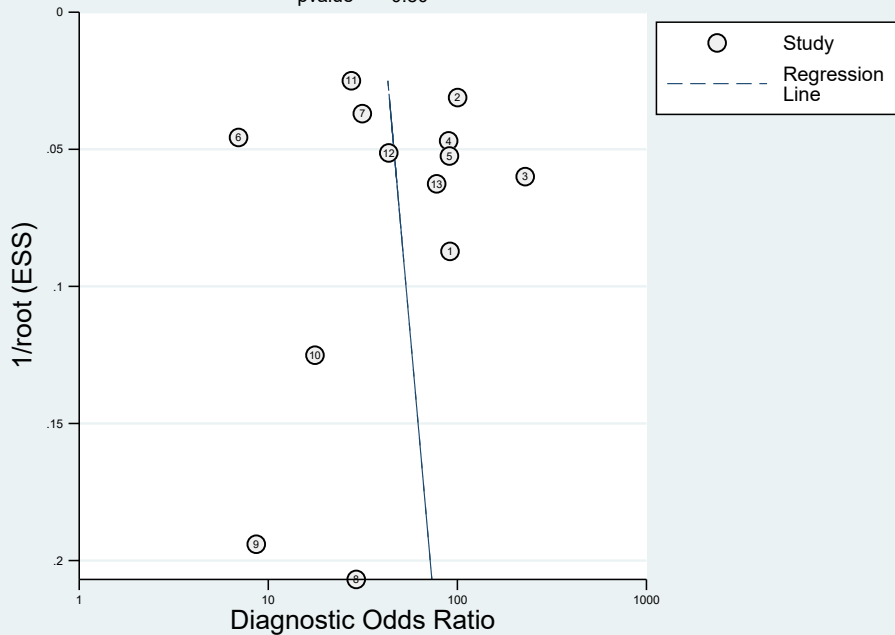

Supplement: Supplementary file 4 — Supplementary Fig. 4 Deeks’ funnel plot of Emergency Department Assessment of Chest Pain Score‐Accelerated Diagnostic Protocol chest pain risk stratification. ESS, effective sample size. [file CLC-46-1303-s007.pdf]

# Univariable Meta-regression & Subgroup Analyses

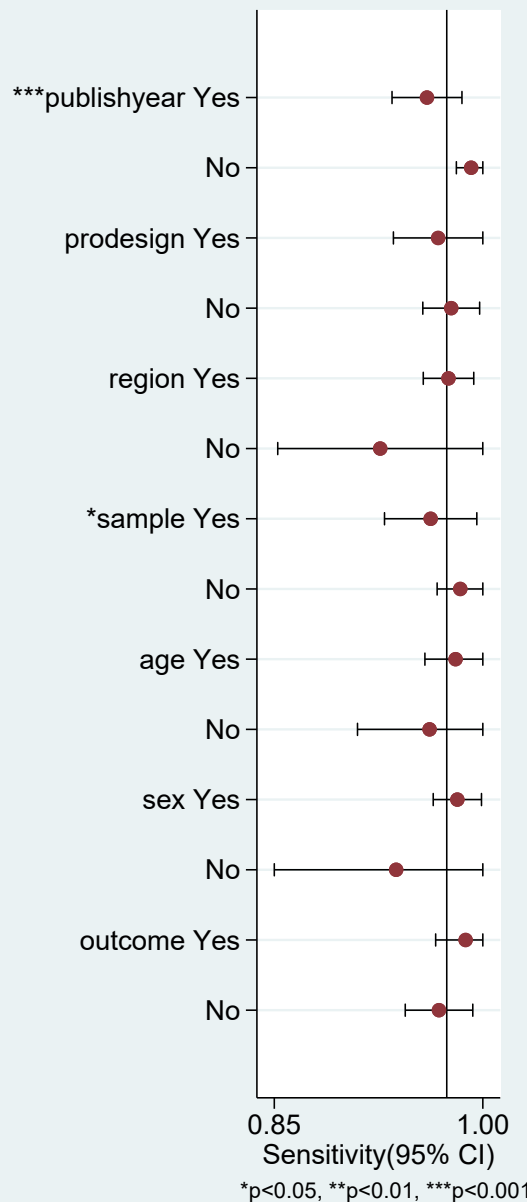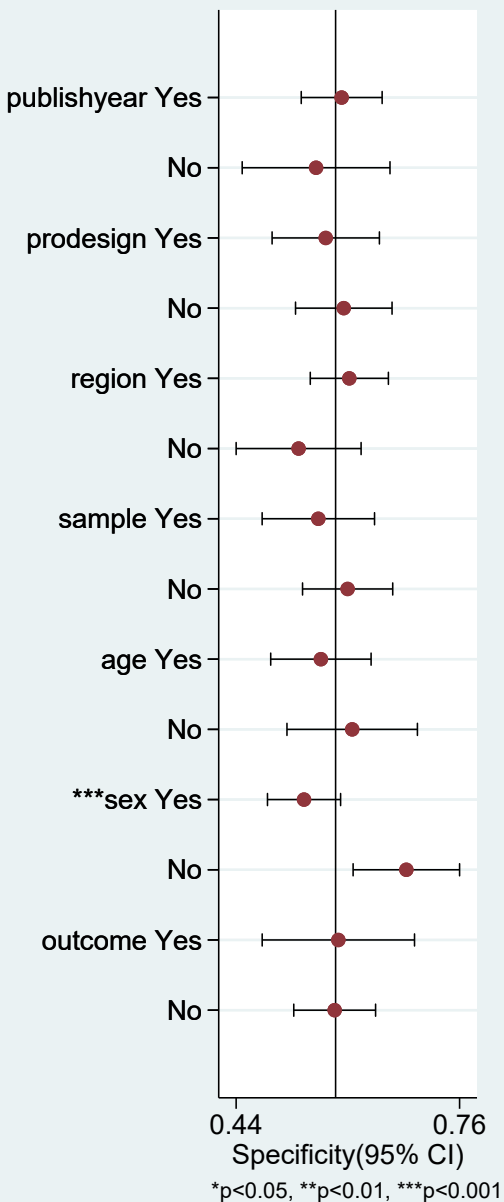

Supplement: Supplementary file 5 — Supplementary Fig. 5 Forest plots for univariate meta‐regression and subgroup analyses. CI, confidence interval; I2, I2 tests; I2lo, I2 tests (low values); I2hi, I2 tests (high values); LRTChi2, χ2 tests. [file CLC-46-1303-s006.pdf]
