# Supplementary material for: A systematic review of the applicability of emergency department assessment of chest pain score‐accelerated diagnostic protocol for risk stratification of patients with chest pain
Source: Clin Cardiol. 2023 Aug 18;46(11):1303–9. doi: 10.1002/clc.24126 (PMC10642332; doi:10.1002/clc.24126)
Supplement: Supplementary file 6 — Supporting information. [file CLC-46-1303-s002.doc]

Supplementary table 1. Emergency department assessment of chest pain score-accelerated diagnostic protocol

| **Clinical Characteristics** | **Score** |
| --- | --- |
| **a)Age**  18 -45  46 -50  51 -55  56 -60  61 -65  66 -70  71 -75  76 -80  81 -85  86+ | +2  +4  +6  +8  +10  +12  +14  +16  +18  +20 |
| **b)Male sex** | +6 |
| **c)Aged 18–50 years and either:**  (i) known coronary artery disease or  (ii)≥3 risk factors  dyslipidemia,diabetes,hypertension,current smoker, or family history of premature coronary artery disease | +4 |
| **d)Symptoms and signs**  Diaphoresis  Radiates to arm or shoulder  Pain occurred or worsened with inspiration  Pain is reproduced by palpation | +3  +5  -4  -6 |
| **Criteria for low risk** | (i)Total score <16 points  (ii)No new ischemia on ECG  (iii)0h and 2h troponin levels below 99th percentile upper reference limit of the assa |
| **Recommendation**:*Low-risk patients were deemed safe for discharge to early outpatient follow-up investigation (or to proceed to earlier inpatient testing).* | |
