# Supplementary material for: A systematic review of the applicability of emergency department assessment of chest pain score‐accelerated diagnostic protocol for risk stratification of patients with chest pain
Source: Clin Cardiol. 2023 Aug 18;46(11):1303–9. doi: 10.1002/clc.24126 (PMC10642332; doi:10.1002/clc.24126)
Supplement: Supplementary file 7 — Supporting information. [file CLC-46-1303-s004.doc]

Supplementary table 2. Comparison of the original study findings of various accelerated diagnostic protocols and results of this study

| ADP | Sensitivity | Specificity | Percent low-risk | 30-day MACE in low-risk patients |
| --- | --- | --- | --- | --- |
| ASPECT-ADP | 99.3% | 11.0% | 9.8% | 0.9% |
| Vancouver chest pain rule | 98.8% | 32.5% | 25.7% | 1% |
| ADAPT-ADP | 99.7% | 23.4% | 20% | 0.25% |
| mADAPT-ADP | 99.2% | 48.7% | 41.5% | 0.8% |
| TRUST-ADP | 99.0% | 44.1% | 39.8% | 0.26% |
| The Heart-pathway | 100% | 83.1% | 82.1% | 0% |
| EDACS-ADP (original) | 99.0% | 49.9% | 57.6% | 0.98% |
| EDACS-ADP (combined） | 97% | 58% | 52.7% | 0.89% |

ADP: accelerated diagnostic protocol; MACE: major adverse cardiovascular events; ASPECT-ADP: Asia-Pacific Evaluation of Chest Pain Trial Accelerated Diagnostic Protocol; ADAPT-ADP: 2-Hour Accelerated Diagnostic Protocol to Assess Patients With Chest Pain Symptoms Using Contemporary Troponins as the Only Biomarker; mADAPT-ADP: 2-Hour Accelerated Diagnostic Protocol to Assess Patients With Chest Pain Symptoms Using High-Sensitivity Troponins as the Only Biomarker; TRUST-ADP: Triage Rule-Out Using High-Sensitivity Troponins Accelerated Diagnostic Protocol; HEART Pathway: the History, Electrocardiogram, Age, Risk Factors, Troponin; EDACS-ADP: Emergency Department Assessment of Chest Pain Score-Accelerated Diagnostic Protocol; Percent low-risk: Proportion of classified as low risk in all patents emergency patients with chest pain
